# Antegrade Elastic Intramedullary Nailing Insertion Technique Results in Higher Incidence of Symptomatic Implants in Pediatric Ulnar Fractures

**DOI:** 10.5435/JAAOSGlobal-D-20-00065

**Published:** 2020-06-01

**Authors:** Taylor R. Johnson, Andrew J. Haus, Kush N. Shah, Abraham I. Bankole, Grant D. Hogue

**Affiliations:** From the University of Texas Health Science Center at San Antonio (Dr. Johnson, Dr. Haus, Dr. Shah, Dr. Bankole, Dr. Hogue), San Antonio, TX, and the Harvard University (Dr. Hogue), Boston, MA.

## Abstract

Retrograde and antegrade nailing techniques are the two options available to a surgeon when using elastic stable intramedullary nailing; however, the literature comparing these two nailing techniques is scarce. Thus, we conducted a retrospective review of all pediatric and adolescent ulnar fractures treated with elastic stable intramedullary nailing at our facility. We hypothesize that the clinical outcomes (implant and wound complications) and the time between surgery and radiographic union will be similar for both techniques.

**Methods:**

A retrospective chart review of pediatric ulnar fracture patients treated at our facility was performed. Demographic and health information associated with the injury were collected, and the clinical outcomes of the two techniques were compared.

**Results:**

A total of 53 patients with 54 fractures were included in this study. Antegrade nail insertion was used to treat 59.2% fractures. Radiographic union was achieved in all patients. Nail insertion technique was not associated with postoperative wound complications, time to radiographic union or implant removal, or significant deficits in upper extremity rotation (*P* > 0.05). Antegrade nailing resulted in a symptomatic implantation 3.97 times more frequently than compared with retrograde nailing (*P* = 0.036).

**Discussion:**

Antegrade nailing demonstrates a similar healing profile but higher implant complications compared with the retrograde nailing technique in pediatric ulnar fractures.

The popularity of elastic stable intramedullary nailing (ESIN) or flexible nailing has increased tremendously for the treatment of unstable long bone fractures, including fractures of the forearm, in pediatric patients since the early reports in the 1980s.^1,2,3,4^ ESIN using intramedullary flex nails (IMNs) is usually preferred over other surgical strategies such as plate fixation, external fixations, and pins/screws with plaster.^3,4^ The widespread acceptance of this technique can be attributed to minimally invasive surgery, absence of the need to use casts for postoperative immobilization, shorter operating time and hospital stay, low complication rates, and early recovery of joint motion, resulting in rapid return to physical activity.^3,5^ Moreover, the biomechanical characteristics of the ESIN provide bending, axial, translational, and rotational stability to achieve optimal results.^6^ Flexible nailing provides favorable radiographic and functional results for radial and ulnar diaphyseal forearm fractures in children and adolescents even when radial bow is not anatomically restored^5,7,8^ and in distal metadiaphyseal fractures^9^ and proximal metadiaphyseal fractures, including certain patterns of Monteggia fractures.^10,11^ Thus, ESIN is an attractive strategy for treating ulnar fractures in pediatric and adolescent patients.

Retrograde and antegrade nailing techniques are two options available to a surgeon when using an IMN for the treatment of ulnar fractures with or without radius involvement. Antegrade nailing involves an entry point directly at the tip of the olecranon or slightly lateral from the olecranon at the proximal ulna. Antegrade nailing provides adequate angular and longitudinal stability to the fracture site; however, the placement of an IMN through the olecranon or near may raise provider concerns regarding symptomatic implantation and irritation of the olecranon bursa, potentially leading to olecranon bursitis and surgical site infections. Similarly, potential injury to the distal ulnar physis or the dorsal sensory branch of the ulnar nerve are common concerns associated with retrograde flexible nailing of the ulnar fractures. Thus, the choice of specific nail orientation is often determined by provider preference.

Both flexible nail orientations are well described and commonly used;^12^ however, no study has compared radiographic and clinical outcomes associated with retrograde and antegrade fixation techniques. The aim of this study is to address this gap in knowledge by comparing the time to radiographic union and postoperative wound and implant complications associated with retrograde and antegrade nail orientations in ulnar fractures treated with ESIN. We hypothesize that the clinical outcomes (incidence of implant and wound complications) and time to radiographic union will be similar in ulnar fracture patients treated with antegrade and retrograde nailing techniques.

## Methods

### Patient Selection and Outcome Measures

A retrospective review of all patients younger than 18 years who underwent flexible IMN for an ulnar diaphyseal fracture at our level 1 accredited trauma center between 2006 and 2018 was performed. Patients were stratified into two groups based on the orientation of the nail placement, retrograde or antegrade. Isolated fractures of the ulna shaft and both-bone forearm fractures that include the radius (Galeazzi, Monteggia, and radial shaft) treated with intramedullary flexible nailing were included because the goal of the study is to compare the two fixation techniques. Patients with previous ipsilateral forearm trauma, underlying bone pathology, or patients receiving care through the juvenile detention system were excluded.

Patient demographics including age, sex, body mass index (BMI), race, and ethnicity were recorded. Injury information including ulnar fracture type (open or closed), mechanism of injury, and location of fracture site (proximal, midshaft, or distal) were also collected. The primary outcome measure was time to radiographic union, and secondary outcome measures were notable deficits in the range of motion, presence of physeal injury resulting in documented growth arrest, postoperative wound or implant complications, and timing to implant removal after successful union. Outcomes of forearm rotation were classified using the criteria by Price et al^13^ (Table 3). Patients were followed by our health system for an average of 9 months postoperatively. All data were collected after an Institutional Review Board (IRB) approval was obtained.

### Surgical Procedure

ESIN was performed under general anesthesia, and the nailing orientation used during the procedure was determined by surgeon preference. Blunt-ended flexible titanium nails were used in all cases and a slight bend 2 to 3 cm from the tip of the nail was made to ease the passage through the medullary canal. Retrograde technique involved the passage of flexible nails through a small longitudinal incision on the ulnar border of the distal ulna. Fluoroscopy was used to identify the location of the distal ulnar physis before making an incision. An incision was made over the subcutaneous border of the distal ulna, just proximal to the location of the distal ulnar physis. Blunt dissection was then performed directly to the bone remaining between flexor carpi ulnaris and extensor carpi ulnaris. Blunt dissection of the subcutaneous structures was performed to protect the tendon and dorsal sensory branch of the ulnar nerve. Again, fluoroscopy was used to ensure that the dissection remained approximately 2 cm proximal to the distal physis and a drill with a soft-tissue guide was used to make an entry hole in the distal ulna for the nail passage. Antegrade technique was performed with either direct entry at the tip of the olecranon or a more lateral approach 1 to 2 cm distal from the olecranon tip; our study did not distinguish between the two possible entry points used for antegrade technique. Direct entry technique was performed through a small incision directly over the tip of the olecranon in line with the ulnar shaft. This incision was continued with a knife to make a small split in the triceps to allow nail entry at the proximal end of the ulna in line with the ulnar shaft. A drill with a soft-tissue protector was then used to gain access to the intramedullary nail in preparation for nail passage. A more lateral entry technique used a 2-cm longitudinal incision along the proximal and lateral aspect of the ulna. After incising skin, blunt dissection was used to split the anconeus muscle fibers to expose the lateral cortex of the proximal ulna approximately 2 to 3 cm distal to the olecranon tip. A drill was then used with a soft tissue protector to gain access to the intramedullary canal approximately 2 to 3 cm distal to the tip of the olecranon. In the event that adequate closed reduction could not be obtained, or if the nail could not be passed successfully in two attempts, the fracture site was opened to provide direct exposure of the fracture site and a subsequent reduction was performed to ensure that no more than three attempts of nail passage were performed. After reduction of the fracture, the rod was advanced across the fracture site according to the standard technique. Because many of these cases involved an associated fracture of the radial shaft, the above-described procedure was performed in conjunction with flexible intramedullary nailing of the radius which was performed according to the standard retrograde technique.

Postoperatively, patients were kept nonweight-bearing and placed in a volar splint or soft dressing according to surgeon preference. The patients were seen at 2 weeks after surgery for examination, suture removal, and initiation of early range of motion exercises. Next, the patients were seen at 6 weeks and 10 to 12 weeks postoperatively. At these appointments, a radiograph is used to monitor fracture healing and maintenance of alignment. If patients were doing well with expected fracture healing, they were instructed to follow-up at 9 months, postoperatively, to schedule the removal of hardware. If there were concomitant soft-tissue concerns, poor motion, or other concerns, the patient was followed more closely according to the clinical need and determination of the attending surgeon. At our institution, we prefer to remove the hardware no earlier than 6 months to reduce the risk of refracture. Most commonly, hardware removal is performed at 9 months postoperatively.

### Statistical Analysis

A Shapiro-Wilk test was used to confirm the normality of data. An independent *t*-test or Mann-Whitney *U* test was used to determine the difference between the 2 groups for normal and non-normal distribution, respectively. Specifically, age and BMI were normally distributed while surgical time, time between surgery and radiographic union, and time between surgery and implant removal when stratified by a nail insertion technique were not distributed normally. Time to implant removal stratified by the incidence of wound or implant complications was normally distributed. Furthermore, a chi-squared test was used to determine the association between two qualitative variables. Binomial regression was then used to establish a correlation between the variables. A *P* value of 0.05 was considered statistically significant.

## Results

### Patient Demographics

**Figure 1:**
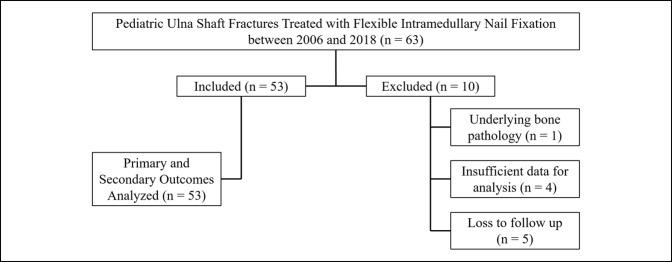
Chart showing the identification of patients for inclusion and analysis.

A Current Procedural Terminology (CPT) code search identified 63 patients treated with flexible IMN for an ulnar shaft fracture. Of these, 10 patients were excluded (Figure 1), one patient had injuries in both arms, resulting in an inclusion of 53 patients with 54 fractures in the study. Overall, race and ethnicity were similar across the 2 groups; 77.4% of all patients were Hispanic or non-Hispanic Caucasians. A patient with injuries in both arms was treated using retrograde nailing in both arms and is counted as a separate record for all analysis except for demographic data calculation (age, sex, BMI, and mechanism of injury). Patient demographics are detailed in Table 1. Our study population consisted of 40 men (75.9%) and 13 women (24.1%) and an average age at surgical management of 10.53 ± 2.56 years. Antegrade nailing technique was used to treat 32 patients, and retrograde technique was used to treat 22 patients. The average follow-up time was approximately 9 months (270.3 days) postoperatively.

**Table 1:** Demographics for Patients Treated Using Intramedullary Flex Nail

	Total	Antegrade	Retrograde	*P* value
Nail fixation technique, n (%)	N = 54	32 (59.2%)	22 (40.8%)	—
Age at surgery, yr, mean ± SD^a^	10.60 ± 2.54	10.75 ± 2.44	10.38 ± 2.72	0.619
Sex^a^				
Male	40 (75.5%)	26 (65.0%)	14 (35.0%)	0.227
Female	13 (24.5%)	6 (46.1%)	7 (53.9%)	
BMI, kg/m^2^, mean ± SD^b^	19.41 ± 4.15	18.74 ± 3.07	20.26 ± 5.18	0.245
Mechanism of injury^a^				
Motor vehicle crash	4 (7.5%)	4 (100.0%)	0 (0.0%)	0.093
Motor vehicle versus pedestrian crash	1 (2.0%)	0 (0.0%)	1 (100.0%)	
Fall (standing)	20 (37.7%)	14 (70.0%)	6 (30.0%)	
Fall (height)	21 (39.6%)	9 (42.9%)	12 (57.1%)	
Other	7 (13.2%)	5 (71.4%)	2 (28.6%)	
Fracture type				
Open	23 (42.6%)	15 (65.2%)	8 (34.8%)	0.443
Closed	31 (57.4%)	17 (54.8%)	14 (45.2%)	
Ulna fracture site				
Distal	16 (29.6%)	10 (62.5%)	6 (37.5%)	0.467
Midshaft	37 (68.5%)	22 (59.4%)	15 (40.6%)	
Proximal	1 (1.9%)	0 (0.00%)	1 (100%)	
Reduction type				
Open	28 (51.8%)	14 (50.0%)	14 (50.0%)	0.151
Closed or percutaneous	26 (48.2%)	18 (69.2%)	8 (30.8%)	
Gustillo Anderson grade, open				
Type 1	17 (73.9%)	11 (64.7%)	6 (35.3%)	0.931
Type 2	6 (26.1%)	4 (66.6%)	2 (33.4%)	
Type 3	0	0 (0.0%)	0 (0.0%)	
Type 4	0	0 (0.0%)	0 (0.0%)	
Type 5	0	0 (0.0%)	0 (0.0%)	
Radius involvement?				
Yes	52 (96.3%)	32 (61.5%)	20 (38.5%)	0.082
No	2 (3.7%)	0 (0.00%)	2 (100%)	
Surgical time, median	83.00	89.50	74.00	0.110

a n = 21 for patients treated with retrograde nailing to account for 1 patient with injuries in both arms.

b n = 27 for patients treated with antegrade nailing and n = 21 for patients treated with retrograde nailing.

### Surgical Technique

**Figure 2:**
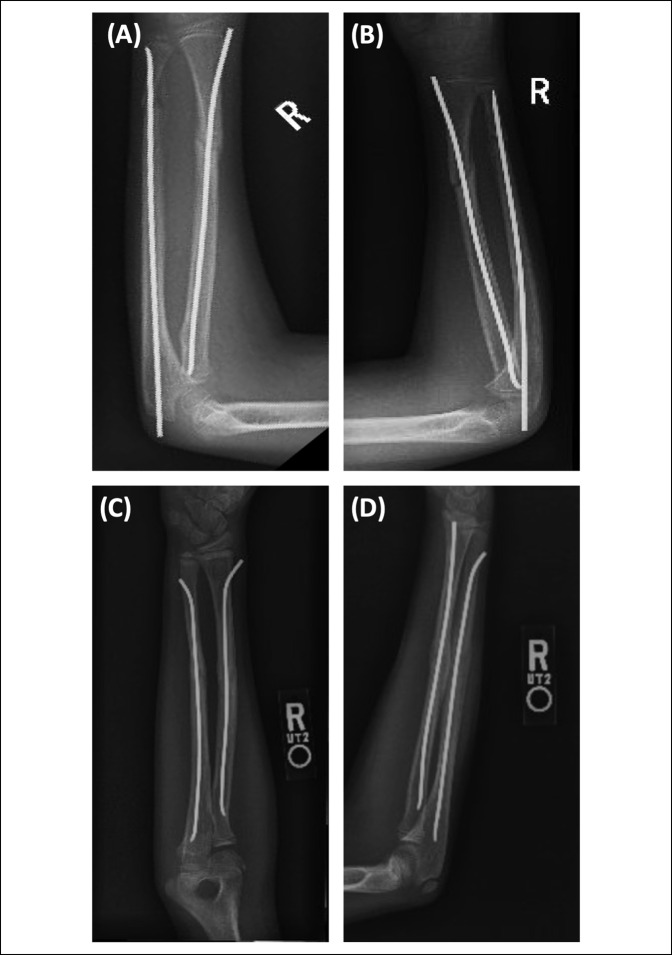
Radiograph of both-bone forearm fracture fixated with (**A** and **B**) antegrade intramedullary nailing of the ulna and retrograde nailing of the radius and (**C** and **D**) retrograde intramedullary nailing of both the ulna and radius in a skeletally immature patient.

Representative images of AP and lateral radiographs of patients treated with IMN are depicted in Figure 2. Figure 2, A and B show AP and lateral radiographs of the ulna treated with antegrade flexible nail, whereas Figure 2, C and D show AP and lateral radiographs of retrograde nail insertion. A portion of the nail is typically left outside, as shown in the radiographs for both techniques, to facilitate later removal. However, we think the prominence of the nail in antegrade nailing results in frequent irritation because of its anatomic location and is observed virtually with all other types of olecranon fixations. Similar prominence of the nail in retrograde technique, however, may result in less implant-related complications associated with irritation because of the oblique insertion angle and anatomic location.

### Radiographic and Clinical Outcomes

Radiographic union, determined by the callus formation and absence of visible fracture lines, was achieved for all fractures included in this study. Nail orientation did not affect the time to radiographic union (*P* = 0.964, Table 2); average time to radiographic union was 12.07 ± 6.43 weeks (median time: 10.50 week) for all patients. In addition, IMN orientation did not affect the incidence of postoperative physeal injury (*P* = 0.769). Two polytrauma patients, one treated with antegrade and the other with retrograde nailing, who sustained both-bone forearm fractures at the metadiaphyseal junction were reported to have injury of the ulnar physis and subsequent growth arrest of the ipsilateral forearm. The incidence of growth arrest in these patients is likely because of the severity and location of injury rather than surgical complications. Significant deficits (greater than 15°) in the upper extremity rotation were not associated with nail orientation technique (*P* = 0.083). Patients were classified into excellent, good, fair, or poor outcomes for forearm rotation based on the criteria established by Price et al^13^ Excellent or good results were obtained in over 95% of patients with both techniques (Table 3). Only one patient treated with retrograde nailing technique for a GA1 open both-bone forearm fracture demonstrated fair results in forearm supination and protonation; however, this patient had developed a concurrent flexion contracture postoperatively because of injury. Finally, despite the complications associated with open fractures, these patients demonstrated comparable time to union (*P* = 0.404) and final range of motion (*P* = 0.902) with closed fracture patients.

**Table 2:** Comparison of Clinical Outcomes in Patients With Ulnar Fractures Treated Using Antegrade or Retrograde IMN Technique

	Antegrade (n = 32)	Retrograde (n = 22)	*P* value
Time to radiographic union			
Mean ± SD	11.7 ± 5.4 wk	12.6 ± 7.8 wk	—
Median	10.50 wk	10.00 wk	0.964
Postoperative wound complications (n, %)	5 (15.6%)	4 (18.2%)	0.804
Postoperative implant complications (n, %)	15 (46.8%)	4 (18.2%)	0.036
Implant removal (n, %)	22 (68.7%)	15 (68.2%)	0.965
Time to implant removal			
Mean ± SD	28.7 ± 10.0 wk	28.9 ± 13.9 wk	—
Median	32.0 wk	28.0 wk	0.637

**Table 3:** The Number of Patients Demonstrating Excellent, Good, or Fair Outcomes for Forearm Rotation After Treatment According to the Criteria Established by Price et al^13^

Outcomes	Symptoms	Loss of Rotation	No. of Patients
Excellent	No complaints with strenuous activity	<15°	Antegrade: n = 29 (93.5%) Retrograde: n = 16 (72.7%)
Good	Mild complaints with strenuous activity	15°-30°	Antegrade: n = 3 (6.5%) Retrograde: n = 5 (22.7%)
Fair	Mild complaints with daily activities	31°-90°	Antegrade: n = 0 (0.0%) Retrograde: n = 1 (4.6%)
Poor	All other results	>90°	—

### Incidence of Wound and Implant Complications

Nail insertion technique was not associated to postoperative wound complications (*P* = 0.804, Table 2); a total of nine postoperative wound complications, including three superficial infections, two nerve injuries, 1 wound dehiscence, and three patients with delayed wound healing of the incision site, were reported. A significant correlation was established between nail orientation and the incidence of implant complications at the insertion site (χ^2^ = 4.95, *P* = 0.036). Implant complications were characterized by incidence of IMN irritation at the insertion site secondary to nail prominence, olecranon bursitis, and implant failure (flexible nail breakage or displacement). The incidence of implant complications was 3.97 times (95% confidence interval, 1.097 to 14.378) higher in patients with antegrade compared with retrograde nailing (Table 2). No incidences of implant failure, including nail displacement or breakage were reported. Despite the association between nail orientation and implant complications, nail orientation did not affect the time to radiographic union or implant removal (*P* > 0.05). Next, the impact of age on incidence of implant complications was also investigated. When stratified into 2 groups, age ≤10 or >10 years, our data did not show any association between age and incidence of complications (*P* = 0.073); 20/26 patients with age ≤10 years and 15/28 patients with age >10 years did not have any implant complications. Finally, the incidence of wound (*P* = 0.389) or hardware (*P* = 0.601) complications was not associated with fracture type.

### Implant Removal

An independent *t*-test indicated a significant difference in the time between surgery and implant removal for patients with and without complications (*P* = 0.043). Patients with postoperative wound complications had their implants removed markedly earlier (mean ± SD: 20.04 ± 10.94 weeks, median: 16 weeks) compared with patients without wound complications (mean ± SD: 30.82 ± 10.87 weeks, median: 32 weeks). Furthermore, a significant correlation was established between the incidence of wound complications and time to implant removal (χ^2^ = 5.602, *P* = 0.018). Incidence of implant complications was not found to affect the time to implant removal in these patients (*P* = 0.100). Nail orientation was not associated with the rate of implant removal (*P* = 0.965, Table 2) or time to implant removal (*P* = 0.637, Table 2).

## Discussion

Antegrade and retrograde nailing techniques are commonly used and have unique advantages and pitfalls associated with each technique. For instance, antegrade nailing is often preferred because of its relative simplicity, whereas retrograde technique allows for easier intraoperative imaging. Furthermore, the choice of the technique may also depend on the fracture location (proximal/distal/middle third). Several studies have examined complications associated with flexible nail fixation in pediatric and adolescent fractures. These studies have primarily focused on the incidence of these complications such as compartment syndrome, loss of fixation, malunion, and nonunion without reference to the specific technique used for nail passage.^14-16^

In our study, we compare the antegrade and retrograde nailing approach for treatment of ulnar fractures. The surgical time for the two techniques was similar for our surgeons (Table 1) and ranged between 30 and 674 minutes, which is considerably higher than the time reported in the literature.^17^ Overall, the 12 patients with surgery time longer than 120 minutes had complicated injuries associated with polytrauma, open fractures, or mechanisms of injury, such as fall or motor vehicle crash, resulting in longer surgical times stemming from the multiple procedures being performed under the same anesthetic. Of these 12 patients, seven patients were treated using retrograde nailing and five patients were treated using antegrade nailing. Thus, the increased time is not associated with the technique but with the complexity of the injury and the multiple procedures being performed concurrently. All patients achieved radiographic union in comparable time, indicating that flexible nail orientation does not affect the rate of union or the time required to achieve a union, regardless of the location or type of fracture. In addition, the time to union reported by us is comparable with the published reports.^18^ The successful use of both retrograde and antegrade nailing techniques for ulna fractures at all locations (proximal, middle, and distal) with no difference in clinical or radiographic outcomes indicate that both techniques can be used proficiently for ulna fractures at all levels. Similarly, no technique preference was noted for the type of fracture (Table 1), and the time to union was independent of fracture type (open versus closed). The overall range of motion in our patients was also found to be comparable with the published reports; 83.1% and 0% of our patients had excellent and poor outcomes, respectively, compared with the published reports of 85.1% excellent and 13.2% poor outcomes.^17^ Finally, none of the patients revisited our clinic for a refracture after successful union.

Retrograde nailing was significantly associated with lower rates of implant complications compared with antegrade nailing, but no association was observed between the nailing technique and incidence of wound complications. These implant complications seemed to be unanimously related to local implant irritation at the insertion site. In addition, conflicting reports have been published associating protruding tip to skin irritation and bursitis. Rokaya et al^19^ attributed increased bursitis and skin irritation to protruding nail tip, which was left out of the cortex by 4 to 5 mm to facilitate removal, whereas Kelly et al^20^ did not experience a notable difference between the buried and exposed tips. Our surgeons leave a protruding nail tip in all patients regardless of the technique used, minimizing the possibility of selective incidence of complications associated with nail tip protrusions in a particular group. Age is also known to affect the incidence of implant complications and time to achieve union in patients treated with IMN; patients older than 10 years demonstrate higher incidence of complications and delayed union.^15,17^ In our patients, age was not significantly (*P* = 0.073) associated with the incidence of implant complications in our patients. Moreover, the time to radiographic union (*P* = 0.964) and implant removal (*P* = 0.958) was comparable for the 2 age groups. Thus, in our population, age did not affect implant complications, and both nailing techniques demonstrated comparable outcomes regardless of age.

The time to implant removal from index surgery was comparable for the two nailing techniques; however, the presence of wound complications, but not implant complications, expedited hardware removal. The minimum recommended time, in the literature, for implant removal is 16 weeks.^21,22^ This recommendation is based on typical fracture healing time with the goal of minimizing complications associated with early hardware removal, primarily, refracture. At our institution, we prefer to wait at least 6 months (24 weeks) before implant removal. This allows ample time for fracture healing and remodeling while still removing hardware before it becomes difficult to remove because of bony overgrowth. In our patients, implants were removed earlier than 16 weeks in five patients, of which two demonstrated both hardware and wound complications, one patient each demonstrated wound and implant complications only, and one patient experienced stiffness. Such early removal of nails because of complications has been previously reported.^23^ The overall complication rate of 26% in our patient population is comparable with the 29% and 34% reported by Freese et al^24^ and Nisar et al,^3^ respectively. Furthermore, none of our patients reported a loss of reduction, radial shortening, refracture, or required a second surgical procedure because of complications. Finally, the fracture type (open or closed) did not affect any outcomes, including incidence of wound or hardware complications, and final range of motion.

This study is the first of its kind to compare antegrade and retrograde intramedullary nailing techniques for fixation in pediatric and adolescent ulna fractures; however, we do recognize that this study is not without limitations. Our study is retrospective in nature and as such raises concerns for potential selection bias secondary to loss of follow-up and collection of data from a single level 1 trauma center. In addition, confusion bias stemming from other variables related to the outcome, such as differing levels of activity, cannot be accounted for in a retrospective chart review. The study included only 54 fractures despite the long study period of 12 years. In addition, the low sample size can also affect the power of statistical tests used in the study. However, the promising results from this study should be used as a springboard to develop a prospective study and compare these fixation techniques and the associated clinical differences in more detail. Moreover, only two of our patients did not have radius involvement, which did not allow us to exclusively evaluate the outcomes specific to ulna IMN. Our analysis also did not consider the material of the nail (stainless steel or titanium) as a cofactor. We have demonstrated comparable surgical time but did not compare the fluoroscopy time for the two nailing techniques. Antegrade nailing technique allows for two starting points, olecranon tip, and a more lateral starting point. A more lateral starting point theoretically can decrease the incidence of local implant irritation and nail prominence. A subgroup analysis of patients treated with antegrade nailing to identify the effect of starting point was not performed. The average follow-up time of 9 months for our patients is shorter than the ideal 1-year postoperative follow-up; however, we feel that our follow-up time is representative of common practice because children are not typically followed long term for diaphyseal ulnar fractures after healing has occurred. In addition, the outdated records limit our ability to contact patients and collect information on further complications, which is a limitation of the study design. As our study's primary focus was to analyze fracture healing and potential complications associated with IMNs, all of our patients, except those lost to follow-up, were all followed to the point of fracture healing or subsequent implant removal, which is preferred by most patients.

This study demonstrates a similar healing profile but lower implant complications for ulna fractures treated with retrograde nailing when compared with antegrade nailing in pediatric patients, refuting part of our hypothesis. Radiographic union was achieved in all patients, and the nailing technique, age, and type of fracture did not affect the time to union or hardware removal in these patients. The impact of nailing technique on implant complications does not translate to increased time to union or implant removal. Finally, the nailing technique was not associated with wound complications; however, incidence of wound complications was found to expedite the hardware removal.
